# From Consensus Through Prioritization: A Delphi Method, Clinical Audit, and Analytic Hierarchy Process Framework for Quality Improvement in Primary Diabetes Care

**DOI:** 10.1177/21501319261462640

**Published:** 2026-06-18

**Authors:** Ansif Pallath Majeed, Niyaz Panakaje, Shajitha Thekke Veettil, Kiran Harikumar, Saurav Sathish Kumta, Hanan Al Mujalli, Sharifullah Khan, Abdul Ali Shah, Noora Jassim AlKubaisi

**Affiliations:** 1 219141 Yenepoya (Deemed to Be University), Mangalore, Karnataka, India; 2 Department of Clinical Affairs, 420951 Primary Health Care Corporation, Doha, Qatar; 3 Yenepoya Research Centre for Finance and Entrepreneurship Development (YEN-REFINED), 219141 Yenepoya (Deemed to Be University), Mangalore, India

**Keywords:** Type 2 Diabetes Mellitus, primary care settings, quality improvement, clinical audit, delphi technique, decision making

## Abstract

**Background:**

Type 2 diabetes mellitus (T2DM) affects approximately 17% of adults in Qatar, making improved primary care management a national health priority. Despite established guidelines, significant gaps persist between recommended care and real-world practice, particularly in preventive and patient-centered services. To address this, a structured, data-driven quality improvement (QI) framework was implemented.

**Methods:**

We conducted a mixed-methods, pre-implementation QI study across 31 primary health care facilities in Qatar. The three-phase approach included: (1) a Delphi consensus with 65 primary care physicians to validate 17 key performance indicators (KPIs) for T2DM care; (2) a retrospective audit of 450 adult T2DM patient records to assess adherence to each KPI; and (3) prioritization of improvement interventions using the Analytic Hierarchy Process (AHP), based on clinical impact (50%), feasibility (30%), and urgency (20%).

**Results:**

The Delphi panel reached unanimous consensus on all 17 KPIs (median importance rating 5/5), covering preventive care, metabolic monitoring, therapy management, and patient education. The audit revealed high adherence to laboratory-based indicators (∼82%) but low performance in preventive and patient-centered care: ASCVD risk assessment (19.6%), foot examinations (24.0%), and individualized HbA1c goal documentation (1.3%). KPI adherence varied widely across centers (e.g., foot exam rates ranged from 0% to 95.6%). AHP analysis identified five top-priority gaps accounting for 63% of total priority weight: ASCVD risk assessment (16%), foot examinations (16%), lifestyle education (12%), follow-up HbA1c testing (10%), and individualized HbA1c goal-setting(9%).

**Conclusion:**

This study highlights critical deficits in preventive and patient-centered diabetes care in primary care facilities. By integrating expert consensus, real-world performance data, and structured prioritization through the Delphi–AHP framework, we developed a robust, evidence-based model for QI planning. Importantly, involving frontline clinicians in both KPI selection and intervention prioritization fostered ownership, contextual relevance, and alignment with clinical realities. This participatory approach enhanced the credibility and feasibility of proposed interventions and laid a strong foundation for sustainable practice change.

## Key Messages

### What Is Already Known on This Topic

• Type 2 diabetes mellitus is highly prevalent in Qatar, and improving the quality and consistency of primary care diabetes management is a national health priority.
• While clinical guidelines for T2DM are well established, gaps persist between recommended care and real-world practice, particularly in preventive and patient-centered domains.
• International frameworks emphasize structured, data-driven approaches in primary care to strengthen diabetes management.

### What This Study Adds

• This study demonstrates the use of a structured Delphi–Analytic Hierarchy Process (AHP) framework to systematically validate diabetes care KPIs and prioritize improvement interventions.
• Although laboratory monitoring showed high adherence, substantial gaps were identified in cardiovascular risk assessment, foot examinations, and individualized glycemic targets, with wide inter-facility variation.
• The AHP approach enabled objective prioritization of improvement actions based on clinical impact, feasibility, and urgency, ensuring focus on areas with the greatest potential benefit.

### How This Study Might Affect Research, Practice, or Policy

• The framework provides a reproducible, evidence-based approach for prioritizing quality improvement initiatives in primary care settings.
• Findings support shifting improvement efforts beyond the clinical management of current problems toward comprehensive risk management and patient-centered care.
• Policymakers and health system leaders can use this approach to guide targeted interventions and resource allocation for diabetes care improvement.

## Introduction

Type 2 diabetes mellitus (T2DM) is a major global health concern, and its prevalence continues to rise. The Middle East and North Africa region has one of the highest diabetes rates in the world (approximately 15–16% of adults, compared to about 10% globally) and is projected to see further increases by 2045.^1^ Qatar, in particular, stands out with a very high burden of T2DM recent estimates indicate that roughly 17–18% of Qatari adults have diabetes, a figure that could nearly double by 2050 in the absence of effective interventions.^2^ This growing epidemic has made improving the quality and consistency of diabetes care a national health priority; Qatar established a comprehensive National Diabetes Strategy (2016–2022) focused on strengthening primary care management, prevention, and patient support.^3^ Despite well-developed services, significant gaps persist between recommended care and real-world practice. For example, a recent study found that about 86% of adults with diabetes in Qatar had above-target HbA_1_c levels (HbA_1_c >7%), underscoring the challenge in translating clinical control goals into routine practice.^4^ This shortfall highlights the need for more effective quality improvement (QI) approaches to ensure that evidence-based diabetes care is delivered consistently.

High-quality primary care facilities are the cornerstone of Qatar’s strategy to combat T2DM. Comprehensive primary care is delivered through a nationally coordinated network of publicly funded health care facilities distributed across the country. These facilities, all accredited to international standards, serve as the frontline for diabetes management, providing early detection, regular monitoring, and preventive services in accessible community settings.^4^ Furthermore, clinical practice guidelines for T2DM are well established both internationally and in Qatar, defining evidence-based care targets and interventions.^5,6^ Current standards (e.g. from the American Diabetes Association and Qatar’s national guidelines) recommend a multifaceted approach encompassing glycemic control, risk factor management, complication screening, and patient education.^5,6^ In practice, however, there is often a notable gap in these areas. Many patients do not receive all recommended preventive and patient-centered interventions, such as annual foot and eye examinations or structured self-management support, without dedicated QI efforts.^7^ Clinical audits and studies worldwide have documented these deficiencies, revealing that important measures for instance, regular foot checks, retinal screenings, or lifestyle counseling are frequently underperformed in routine care.^7^ Similar patterns have been observed in Qatar’s primary care system, where adherence to certain preventive services remains suboptimal, contributing to preventable complications and poor risk-factor control. These care gaps persist despite clear guideline recommendations; for example, standards emphasize timely initiation of metformin as first-line therapy and prompt treatment intensification (including adding medications or insulin) when glycemic targets are not met, to avoid therapeutic inertia.^8^ Likewise, guidelines endorse individualized HbA_1_c targets and periodic goal-setting with patients to ensure treatment is tailored to patient context.^8^ The discrepancy between “knowing” and “doing” in diabetes care particularly in preventive and patient-centered domains underscores the urgency of implementing systematic QI mechanisms to bridge these gaps.

Effective prioritization of QI efforts is critical, given the broad spectrum of potential interventions and limited resources. Structured, transparent frameworks that link best-practice evidence, expert consensus, and local performance data are recommended to guide decision-makers in selecting high-impact improvement areas. In this context, approaches like the Delphi method (for achieving expert consensus) and multi-criteria decision analysis (for ranking priorities) have gained attention as tools to bring clarity and objectivity to QI planning. However, these methodologies had not previously been applied in an integrated way to diabetes care in Qatar’s primary health system.

Study Objectives: To address the challenges above, this study developed and tested a novel “Delphi–AHP” framework for advancing quality improvement in primary diabetes care. The objectives were: (1) to engage local clinical experts in a Delphi consensus process to identify priority key performance indicators (KPIs) of T2DM care quality in Qatar’s primary care system; (2) to evaluate current performance on the agreed KPIs through a structured clinical audit of primary care clinics, thereby pinpointing major gaps between recommended and actual care; and (3) to apply the Analytic Hierarchy Process (AHP) a multi-criteria decision-making tool to systematically prioritize quality improvement interventions based on the Delphi consensus and audit findings. By combining expert consensus with quantitative performance data and explicit priority-setting criteria, this framework aims to ensure that efforts to improve diabetes care are evidence-driven and focused on the areas of greatest need. In the sections that follow, we describe how this Delphi–AHP approach was implemented and discuss its potential to enhance T2DM care quality in Qatar’s primary care settings.

## Methodology

### Study Design and Setting

**Figure 1.**
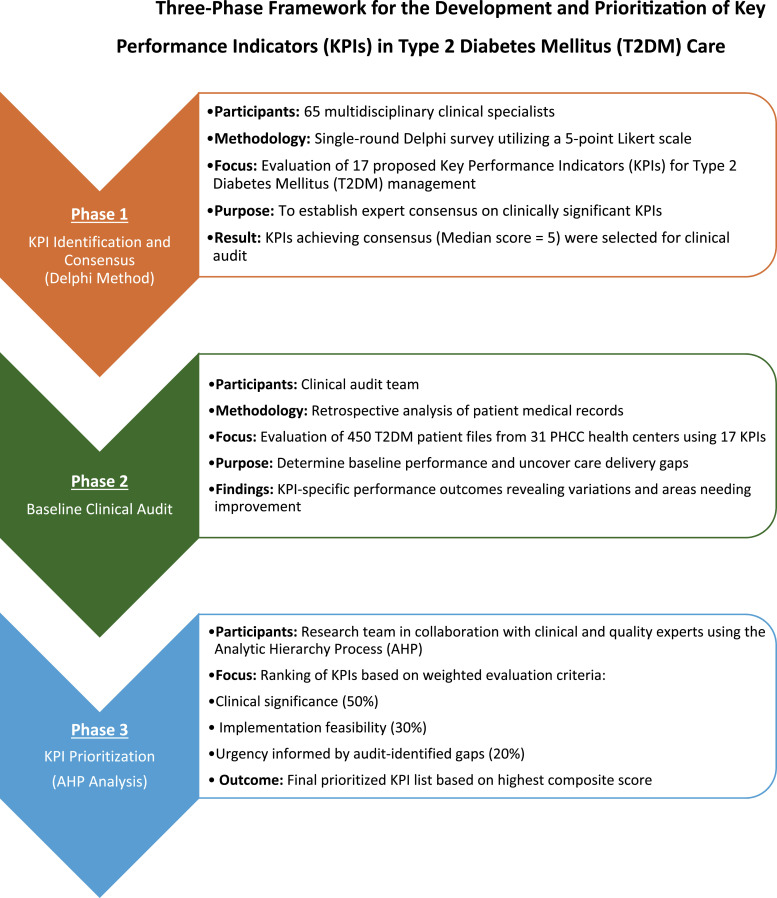
Three-phase framework for the development and prioritization of Key Performance Indicators (KPIs) in Type 2 Diabetes Mellitus (T2DM) care Figure 1 Overview of the three-phase process for developing and prioritizing Key Performance Indicators (KPIs) for Type 2 Diabetes Mellitus (T2DM) care. Phase 1 involved a one-round Delphi survey with 65 multidisciplinary clinical experts who rated 17 candidate KPIs on a 5-point Likert scale to establish consensus on clinical relevance (KPIs with a median score of 5 were shortlisted). Phase 2 comprised a retrospective clinical audit of 450 T2DM patient records across 31 Primary Health Care Corporation (PHCC) health centers to assess baseline performance and identify care gaps. Phase 3 utilized the Analytic Hierarchy Process (AHP) to prioritize KPIs based on weighted criteria: clinical impact (50%), feasibility of implementation (30%), and urgency informed by audit-identified gaps (20%), resulting in a final prioritized list of high-impact KPIs for quality improvement.

We conducted a mixed-methods, pre-implementation quality improvement (QI) study within the primary care facilities. The study followed a structured, three-phase framework designed to identify and prioritize key areas for enhancing type 2 diabetes mellitus (T2DM) care across 31 government-funded primary care centers. The phases included expert consensus-building through a Delphi process to identify and rate 17 key performance indicators (KPIs) for diabetes care; a retrospective clinical audit of 450 adult T2DM patient records to assess baseline adherence to these KPIs and uncover performance gaps across centers; and prioritization of improvement targets using the Analytic Hierarchy Process (AHP), which incorporated expert pairwise comparisons based on clinical impact, feasibility, and urgency. No clinical interventions were implemented or evaluated during the study period. This initiative represents a formative, observational QI assessment aligned with SQUIRE 2.0 guidelines, intended to inform the design and implementation of future interventions. A follow-up re-audit is planned after implementation to evaluate changes in practice and assess the impact of the prioritized interventions. Figure 1 outlines the study framework.

### Phase 1 – Delphi Consensus on KPIs

In Phase 1, we used a modified Delphi technique to achieve expert consensus on a set of 17 candidate key performance indicators (KPIs) for quality diabetes care. These KPI candidates were initially derived from established international and national T2DM clinical practice guidelines (including Qatar’s adapted guidelines based on American Diabetes Association, International Diabetes Federation standards, WHO report of Diabetes etc.) and input from local clinical leadership. The KPIs were chosen to cover critical domains of diabetes management (preventive care, monitoring tests, therapy management, and patient education) in primary care.^9^

### Delphi Panel and Procedure

We identified a panel of 65 experienced primary care physicians (Senior Consultants, Consultants, and Specialists in Family Medicine) across PHCC centers, each with at least 15 years of experience managing chronic diseases (chiefly T2DM). Panelists were invited via email and acquired consent to participate. In the first Delphi round, experts independently rated the importance of each of the 17 proposed KPIs on a 5-point Likert scale (1 = “Not at all important” to 5 = “Extremely important”). Table 2 (below) lists the 17 KPIs by domain. Participants considered each indicator’s relevance to high-quality primary diabetes care when assigning ratings. We defined a consensus a priori as a median rating of 4 or 5 (indicating high importance) across the panel. In this study, all 17 indicators were very highly rated (median score = 5) in the first round, with minimal variability in responses. Because this strong agreement was achieved in Round 1, we concluded that consensus was reached and did not conduct a second round. (Had there been substantial disagreement, for example, a wide range of scores on an item, we would have provided anonymous feedback summaries and conducted further Delphi rounds to resolve differences.) After the single round, the results (aggregate scores and anonymized comments) were shared with the panel for confirmation. The Delphi process thus confirmed the face and content validity of the proposed KPI set for our context. Notably, the expert panel’s endorsement of these indicators also reflected their consistency with recognized standards of diabetes care (e.g. routine cardiovascular risk assessment for patients ≥40, annual foot and eye examinations, etc.).^9,10^

### Phase 2 – Clinical Audit (Baseline Assessment)

In Phase 2, we carried out a retrospective clinical audit to measure current performance on the 17 agreed-upon KPIs and identify gaps in care. We randomly sampled 450 adult T2DM patient records from the 31 primary care facilities (approximately 13–15 records per center) for the period October 2024 to February 2025. This sample size (∼15 charts per clinic) was chosen to ensure representation of all health facilities while providing a snapshot of recent diabetes care practices. Trained clinical auditors reviewed electronic medical records and extracted data for each KPI using a standardized audit tool with explicit definitions for what constituted compliance on each indicator (based on national guideline criteria). For instance, we verified whether an ASCVD risk score was documented for eligible patients (age ≥40), whether a complete foot exam note was present, whether recommended lab tests were ordered in the past year, etc. Lack of documentation for a given indicator was counted as non-adherence to that KPI (a conservative assumption consistent with audit standards). We also assessed inter-rater reliability by double-reviewing 10% of sampled charts; Cohen’s kappa exceeded 0.8 for key variables, indicating high consistency. Any discrepancies were resolved through discussion and re-checking the source data, and periodic quality checks were performed during data collection to ensure accuracy and standardization.

During the audit period, we additionally conducted a patient telephone survey to incorporate patient perspectives (qualitative/subjective data) alongside the chart review (quantitative data). We randomly selected 60 patients from the audited cohort and administered a brief structured questionnaire about their recent diabetes clinic visit. This survey asked patients whether their physician had discussed key care elements (for example, “Did your doctor talk with you about your diabetes symptoms and complications?”, “Did you receive advice about diet and physical activity?”, “Were you told your personal target HbA_1c_ (glycemic goal)?”, and “Overall, are you satisfied with the diabetes care you received?”). Responses were recorded as yes/no, and overall satisfaction was rated on a yes/no scale. The survey results were used to triangulate the audit findings, for instance, by checking if patients recalled the same education or goal-setting that clinicians documented, and to highlight any disconnect between provider documentation and patient perception (e.g., the audit showed only 1.3% of charts had a documented HbA_1c_ goal, and similarly only about half of patients recalled having a goal discussion).

The clinical audit revealed the prevalence of each KPI in practice and the variability across centers. In general, we found high adherence to many laboratory-related indicators (e.g. nearly 82% average completion of recommended baseline lab panels) but much lower performance on several preventive and patient-centered processes (detailed results are provided in the Results section). For example, the audit identified that only about 20% of eligible patients had an ASCVD risk score documented, 24% had evidence of a documented foot exam, and just 1% had an individualized HbA_1c_ goal recorded. These data confirmed that even for the indicators that experts deemed important, there were considerable gaps in real-world practice, reinforcing the need for improvement. We regarded the audit findings as a “baseline” assessment of current performance (for internal QI purposes), recognizing that no interventions had yet been introduced. The audit results were immediately fed into the next phase of the study to ensure that prioritization of interventions would be grounded in evidence of which gaps were largest. In this way, the mixed-methods design allowed us to integrate qualitative expert opinion (Delphi) with quantitative performance data (audit), strengthening the overall analysis through triangulation of findings.

### Phase 3 – Prioritization With Analytic Hierarchy Process

In Phase 3, we applied the Analytic Hierarchy Process (AHP), a multi-criteria decision analysis technique, to systematically prioritize which of the 17 KPI areas should be addressed first with quality improvement interventions. The AHP was carried out by a working group consisting of clinical audit champions, all of whom are experienced physicians, clinicians, and clinical audit specialists and auditors including several members from the Delphi panel. We first defined three key criteria for prioritizing improvement needs, informed by literature on healthcare priority-setting and expert consensus: Clinical Impact, Feasibility, and Urgency. Clinical Impact was defined as the expected benefit to patient outcomes if a particular care process is improved (analogous to perceived importance); Feasibility reflected the ease or practicality of implementing an improvement intervention for that area (considering factors like resources, training, and system constraints); and Urgency captured the timeliness or pressing need for action (for instance, an area with a severe performance gap or patient safety risk might be deemed more urgent). Through facilitated discussion and pairwise comparisons among these criteria, the group assigned relative weights of 50% to Clinical Impact, 30% to Feasibility, and 20% to Urgency. This weighting scheme placed the greatest emphasis on patient outcome impact, consistent with recommended practice in healthcare decision-making.

Next, each of the 17 KPIs (the “alternatives” in AHP terms) was appraised with respect to the three criteria. To incorporate the prior Delphi results into the model, the group used the Delphi importance scores as a surrogate for Clinical Impact, effectively assuming that the expert panel’s median importance rating for a KPI reflected its potential impact on patient care. For the Feasibility and Urgency criteria, the working group drew on their expert judgment in light of the audit findings: for example, an indicator that had extremely low compliance in the audit could be judged as having high urgency to address. The group evaluated each KPI’s performance on Feasibility and Urgency through discussion and consensus-based scoring (using a 9-point scale where higher scores indicated greater feasibility or urgency). Pairwise comparisons among the three criteria (Clinical Impact, Feasibility, and Urgency) were performed using Saaty’s standard 1–9 ratio scale, wherein a score of 1 indicates equal importance between two criteria and a score of 9 indicates that one criterion is extremely more important than another. The resulting comparison matrix was used to derive the relative criterion weights (50%, 30%, and 20%, respectively). To verify the internal consistency of the pairwise judgments, the Consistency Ratio (CR) was calculated using the standard AHP formula: CR = CI/RI, where CI is the Consistency Index [(λmax – n)/(n – 1)] and RI is the Random Index for the matrix size. The CR for the criteria matrix was below the accepted threshold of 0.10, confirming acceptable consistency of the expert judgments. All AHP calculations, including derivation of priority weights and consistency testing, were performed using dedicated AHP decision-support software. The software applied Saaty’s eigenvalue method to derive normalized priority vectors and compute the CR automatically, ensuring computational accuracy and methodological consistency throughout the prioritization process. We then entered the criteria weights and the scores for each KPI into the software to compute a composite priority score for each improvement area. This score is a weighted aggregate reflecting each KPI’s relative priority, and the scores were normalized to sum to 1 (100%). Finally, we ranked the KPIs from highest to lowest priority based on these composite scores. In essence, the AHP allowed us to combine information on what should be improved (from the Delphi phase), where the biggest gaps exist (from the audit), and practical considerations (feasibility and urgency) into a single prioritized list of targets for quality improvement. Importantly, because the audit data were shared with the AHP decision-makers, areas with both high clinical importance and poor current performance rose to the top of the priority list. (We report the specific priority rankings in the Results section; broadly, processes such as risk assessment and foot exams which had strong consensus importance but were often missed in practice received the highest priority for intervention, whereas processes that were already done well, like first-line medication prescribing, were ranked lower.) This structured prioritization ensures that subsequent QI efforts can be focused on the “vital few” areas with the greatest potential to improve patient outcomes.^11^

### Data Quality Assurance

To ensure the completeness and accuracy of data collected during the baseline clinical audit, a standardized data abstraction protocol was employed across all 31 primacy care facilities. A structured data abstraction form was developed based on the 17 validated key performance indicators (KPIs), with clear operational definitions aligned with national and international clinical guidelines. All data collectors received formal training on the use of the abstraction tool and on consistent extraction of KPI-related information from the electronic medical record (EMR) system.

To assess inter-rater reliability, a random 10% subset of patient records was independently reviewed by a second auditor. Inter-rater agreement was calculated using Cohen’s kappa statistics, demonstrating high consistency across reviewers. Additionally, random spot checks were conducted by senior auditors to compare extracted data against original EMR entries. Discrepancies were documented, and corrective feedback was provided to ensure uniformity in data capture.

Missing or undocumented data were treated as non-compliant with the respective KPI, in line with standard audit methodology. The proportion of missing data was recorded for each indicator and reviewed during data analysis. These procedures ensured that the audit findings accurately reflected real-world documentation practices and provided a reliable foundation for prioritizing quality improvement interventions.

### Statistical Analysis

Descriptive and inferential statistical analyses were conducted to evaluate adherence to the 17 selected key performance indicators (KPIs) across 31 primary care facilities. Categorical variables (e.g., completion of foot exams, ASCVD risk assessments) were summarized using frequencies and percentages. Continuous variables (e.g., age, HbA1c levels) were summarized using means and standard deviations.

To assess variability in KPI adherence across centers, we used the chi-square (χ^2^) test for independence. This test evaluated whether observed differences in adherence rates were statistically significant across the 31 centers. Paired t-tests were used to compare performance gaps between related indicators (e.g., baseline vs. follow-up HbA1c testing) within the same centers.

Pearson correlation analysis was conducted to explore associations between pairs of KPIs, such as the relationship between lifestyle education and dietitian referrals. Linear regression analysis was used to examine whether adherence to one KPI could predict performance on another. In these models, the dependent variable was the adherence rate to a specific KPI (e.g., dietitian referral), and the independent variables included other related KPIs (e.g., lifestyle education). Regression coefficients (β), 95% confidence intervals (CI), and p-values were reported to describe the direction and strength of associations.

All statistical tests were two-tailed, and a p-value of <0.05 was considered statistically significant.

## Results

### Delphi Consensus on Key Performance Indicators

A Delphi survey involving 65 PHCC clinical experts was conducted to rate 17 proposed key performance indicators (KPIs) for type 2 diabetes mellitus (T2DM) care. All indicators achieved a median importance score of 5 on a 5-point Likert scale (1 = “Not at all important” to 5 = “Extremely important”), indicating strong consensus. The highest-rated indicators by mean score were HbA1c testing (4.93), lifestyle education (4.89), serum creatinine and GFR (4.89), lipid profile (4.86), and both retinal exam referral and urine ketone testing (4.84). These findings reflect strong alignment with international guidelines and emphasize the panel’s prioritization of comprehensive metabolic monitoring and patient education.

Indicators such as ASCVD risk assessment (4.54), foot examination (4.54), and HbA1c goal setting (4.56) also achieved full consensus but demonstrated greater variability in individual ratings. Table 1 shows expert ratings of candidate KPIs, while Table 2 lists Diabetes Care KPIs by domain.

**Table 1.** Expert Ratings of Candidate Key Performance Indicators (KPIs) for Type 2 Diabetes Mellitus (T2DM) Care Based on Delphi Survey (n = 65)

Indicator	mean	median	min	max
ASCVD Risk Assessment	4.54	5	1	5
Laboratory Investigations: HbA1c	4.92	5	4	5
Laboratory Investigations: Lipid profile (total cholesterol, HDL, LDL, triglycerides)	4.85	5	4	5
Laboratory Investigations: Serum creatinine and calculated GFR	4.89	5	4	5
Laboratory Investigations: Liver function tests (ALT, AST)	4.52	5	2	5
Laboratory Investigations: Albumin/creatinine ratio	4.70	5	3	5
Laboratory Investigations: TSH (if dyslipidemia or women >50 years)	4.54	5	3	5
Laboratory Investigations: Urine ketones (if hyperglycemia >250 mg/dL)	4.84	5	1	5
Laboratory Investigations: Serum potassium (for patients on ACE inhibitors, ARBs, or diuretics)	4.73	5	3	5
HbA1c Goal Setting: Individualized HbA1c target documented	4.56	5	1	5
Initial general foot examination	4.5	5	2	5
Referral to eye specialist for initial retinal assessment	4.8	5	3	5
Pharmacologic Management: Metformin prescribed as first-line therapy	4.40	5	1	5
Pharmacologic Management: Timely escalation to dual therapy when indicated	4.78	5	3	5
Lifestyle Education	4.89	5	3	5
Dietitian Referral	4.57	5	1	5
Follow-Up and Lab Monitoring	4.77	5	3	5

Table 1 summarizes each indicator’s mean, median, and range. The highest mean scores were for HbA1c Laboratory Investigations (4.93), Lifestyle Education (4.89), Serum Creatinine and GFR (4.89), Lipid Profile (4.86), and both Retinal Assessment Referral and Urine Ketone Testing (4.84), highlighting strong adherence to international guidelines in monitoring and education.

**Table 2.** Key Performance Indicators (KPIs) for Diabetes Care, by Domain (as Identified via Delphi Consensus)

Domain	Key Performance Indicators (KPIs) for T2DM care
**Preventive Care (Screenings & Risk Assessment)**	– **Atherosclerotic cardiovascular risk assessment** (conduct risk stratification for patients ≥40 years old)
	– **Comprehensive diabetic foot examination** (foot inspection with neurologic & vascular assessment, especially at diagnosis and annually thereafter)
	– **Referral for retinal (eye) examination** (dilated eye exam at diagnosis and at least yearly for early detection of retinopathy)
**Laboratory Investigations (Monitoring tests)**	– **HbA**_**1c**_ **testing** at recommended intervals (at least twice yearly if at goal, quarterly if not at goal, to monitor glycemic control)
	– **Fasting lipid profile** (annual cholesterol panel for cardiovascular risk management)
	– **Renal function tests** (annual serum creatinine and estimated GFR to assess kidney function)
	– **Urine albumin-to-creatinine ratio** (annual screening for diabetic nephropathy)
	– **Liver function tests** (baseline ALT/AST to monitor for hepatic comorbidities or medication effects)
	– **Thyroid function (TSH) test** (screening for hypothyroidism in patients with dyslipidemia or women >50, per guidelines)
	– **Serum potassium monitoring** (for patients on ACE inhibitor/ARB or diuretic therapy, to detect hyperkalemia)
	– **Urine ketone testing** (if marked hyperglycemia, e.g. blood glucose >250 mg/dL, to evaluate for ketoacidosis)
**Pharmacologic Management**	– **Metformin as first-line therapy** (initiating metformin in newly diagnosed T2DM if no contraindications, in line with guidelines)
	– **Timely therapy intensification** (escalation to dual therapy or insulin initiation when glycemic targets are not met, to avoid therapeutic inertia)
	– **Guideline-concordant medications for risk reduction** (prescribing recommended adjunct therapies when indicated – e.g. statins for cardiovascular risk >_^40^ years, ACE inhibitors for albuminuria – in accordance with clinical guidelines)
**Patient Education & Follow-Up**	– **Lifestyle modification counseling** (providing education on diet, physical activity, and self-management strategies)
	– **Referral to dietitian** for Medical nutrition therapy (personalized dietary counseling to support lifestyle changes)
	– **Individualized HbA**_**1c**_ **goal setting** (documenting a personalized glycemic target in the care plan, based on patient’s conditions and shared decision-making)
	– **Scheduled follow-up for glycemic re-evaluation** (arranging timely follow-up visit and repeat HbA_1c_ testing – typically within 3–6 months – to monitor treatment effectiveness)

Table 2 summarizes17 key performance indicators for diabetes care that were established via Delphi consensus, grouped by care domain. These served as the audit criteria in Phase 2. Each KPI reflects an evidence-based practice recommended by diabetes management guidelines and considered important and feasible for quality improvement in primary care. For example, routine ASCVD risk scoring and foot and eye exams are strongly advised by international and national standards to reduce cardiovascular and microvascular complications. Similarly, regular lab monitoring (e.g. HbA_1c_, lipids, renal tests) is critical for guiding therapy, and first-line metformin with timely escalation of treatment is recommended to prevent prolonged hyperglycemia. Finally, patient-centered practices such as providing lifestyle counseling, involving dietitians, and setting individualized glycemic targets are essential for supporting self-management and aligning care with each patient’s needs. The Delphi panel’s unanimous agreement (all KPIs were rated “very important”) indicated that these 17 indicators were collectively seen as a valid and comprehensive representation of quality care in T2DM.

### Clinical Audit Findings

A retrospective audit of 450 adult T2DM patient records across 31 primary health facilities revealed significant variability in adherence to the selected KPIs (Table 2). While laboratory investigations were generally well-performed (mean compliance 81.8%), preventive measures such as ASCVD risk assessment (19.6%) and foot examinations (24.0%) were frequently underperformed. Documentation of individualized HbA1c targets was virtually absent (1.3%).

Chi-square tests demonstrated statistically significant differences in adherence rates across centers for all indicators (p < 0.001). For example, foot exam completion ranged from 0% to 95.6% across centers (χ^2^(30) = 956.4, p < 0.001), and ASCVD risk assessment ranged from 0% to 100% (χ^2^(30) = 912.4, p < 0.001). These disparities reflect substantial inter-center variation; however, we acknowledge that such variation may reflect random inconsistency rather than systematic differences in care delivery.

Paired t-tests were used to compare performance gaps between related indicators. Foot exams lagged behind lab tests by an average of 57.7 percentage points (t(30) = 9.53, p < 0.001), and ASCVD risk assessments were 62% less frequent than baseline labs (p < 0.001). Follow-up HbA1c testing (3–6 months) was also significantly underperformed compared to baseline testing (mean difference ∼40%, t(30) = 8.41, p < 0.001), indicating a drop in care continuity.

Pearson correlation analysis showed limited associations between most indicators. A moderate positive correlation was observed between lifestyle education and dietitian referrals (r = 0.404, p = 0.024), suggesting that patient-centered counseling may be associated with broader care coordination.

Linear regression analysis was conducted to explore predictors of dietitian referral rates (dependent variable). The rate of lifestyle education was a significant predictor (β = 0.524, 95% CI [0.07, 0.98], p = 0.025), while baseline lab completion was not (β = –0.163, p = 0.66). No significant predictors were identified for eye specialist referrals. Table 3 presents a detailed analysis.

**Table 3.** Audit Compliance With Key T2DM Care Indicators Across 31 PHCC Health Centers: Audit Results and Inter-center Variability

Key indicator (Baseline Audit Measure)	Overall mean compliance (±SD)	Range across centers	Heterogeneity (Chi-square^†^ test)
**ASCVD Risk Assessment (age ≥40 at diagnosis)**	19.6% (±31.9%)	0% – 100%	**χ** ^**2**^ **(df=30) = 912.4, p < 0.001**
**Lab Investigations Ordered (baseline labs)**	81.8% (±14.8%)	56% – 99%	**χ** ^**2**^ **(df=30) = 100.8, p < 0.001**
**Foot Examinations Done (initial visit)**	24.0% (±27.5%)	0% – 95.6% ^‡^	**χ** ^**2**^ **(df=30) = 956.4, p < 0.001**
**Eye Specialist Referrals (retinal exam)**	33.8% (±26.4%)	0% – 100%	**χ** ^**2**^ **(df=30) = 579.1, p < 0.001**
**HbA**_**1c**_ **target Documented**	1.3% (±2.4%)	0% – 7%	**χ** ^**2**^ **(df=30) = 295.2, p < 0.001**
**Metformin Compliance (patient adherence)**	70.4% (±30.1%)	18% – 100%	**χ** ^**2**^ **(df=30) = 405.8, p < 0.001**
**Dual therapy Compliance (if indicated)**	76.3% (±28.7%)	0% – 100%	**χ** ^**2**^ **(df=30) = 1018.4, p < 0.001**
**Guideline-appropriate drug Rx**	72.7% (±25.1%)	29% – 100%	**χ** ^**2**^ **(df=30) = 442.8, p < 0.001**
**Lifestyle Education Provided (diet/exercise advice)**	24.1% (±24.7%)	0% – 87%	**χ** ^**2**^ **(df=30) = 284.9, p < 0.001**
**Dietitian Referral (MNT)**	39.0% (±25.0%)	13% – 93%	**χ** ^**2**^ **(df=30) = 410.9, p < 0.001**
**Follow-up HbA**_**1c**_ **Test (3–6 months)**	41.7% (±24.4%)	0% – 87%	**χ** ^**2**^ **(df=30) = 556.1, p < 0.001**

Table 3 The baseline audit revealed substantial variability in compliance with key T2DM care indicators across PHCC health centers. While laboratory investigations showed relatively high compliance (e.g., baseline labs at 81.8%), critical areas such as ASCVD risk assessment (19.6%), foot examinations (24.0%), eye referrals (33.8%), and HbA1c target documentation (1.3%) demonstrated notably low adherence. Significant inter-center heterogeneity was observed for all indicators (p < 0.001), highlighting inconsistent care delivery and opportunities for targeted quality improvement.

### Patient Satisfaction Survey

A follow-up telephone survey of 60 patients with T2DM revealed high overall satisfaction with care (97%). Most patients reported receiving symptom discussions (87%) and lifestyle advice (83%). Only 47% recalled discussions about individualized HbA1c goals, consistent with the low documentation rate observed in the audit.

### AHP-Based Prioritization of Improvement Interventions

The AHP analysis prioritized interventions based on expert pairwise comparisons of the 17 KPIs using three criteria: clinical impact (50%), feasibility (30%), and urgency (20%).

The top five prioritized indicators were ASCVD risk assessment and initial diabetic foot examination (each 16% of total priority weight), followed by lifestyle education (12%), follow-up HbA1c testing (10%), and documentation of individualized HbA1c goals (9%). These five indicators together accounted for 63% of the total priority weight.

Interpretive statements regarding the rationale for lower prioritization of well-adhered indicators (e.g., metformin initiation) and the influence of baseline compliance have been moved to the Discussion section, as requested. Table 3 AHP Criteria and Weights, and Top 5 Prioritized Diabetes Care Interventions. Table 4 shows AHP criteria, weights, and the five top-ranked diabetes care interventions.

**Table 4.** AHP Criteria and Weights, and Top 5 Prioritized Diabetes Care Interventions

AHP evaluation criterion	Weight (%)	Top-ranked improvement area (KPI)	Priority score (%)
Clinical Impact – importance of the improvement on patient outcomes	50%	1. **Atherosclerotic Cardiovascular Risk Assessment** (for eligible patients) – *Top Priority*	16%
Feasibility – ease of implementation given resources and context	30%	2. **Initial Diabetic Foot Examination** (at first visit) – *Top Priority*	16%
Urgency – timeliness/level of current gap in care	20%	3. **Lifestyle Education Provided** (dietary & exercise counseling)	12%
*(–)*	*(–)*	4. **Follow-up HbA1c Testing** (within 3–6 months of baseline)	10%
*(–)*	*(–)*	5. **Individualized HbA1c Goal Documentation** (in patient record)	9%

Table 4 shows the three AHP criteria (Clinical Impact, Feasibility, Urgency) were compared pairwise by the expert panel to determine their weights (50%, 30%, 20% respectively). **Top-ranked improvement areas** are the diabetes care indicators that received the highest AHP global priority scores based on those criteria weights. As shown, “ASCVD Risk Assessment” and “Initial Foot Examination” were both highest priority (each ∼16% of total weight), indicating that interventions to improve these areas should receive the greatest emphasis. “Lifestyle Education,” “Follow-up HbA1c Testing,” and “HbA1c Goal Documentation” were ranked next, with priority scores of ∼12%, 10%, and 9% respectively. These values are derived from the weighted AHP calculations and align with the fact that the first two areas had both high clinical importance and large performance gaps in our audit data. We have included this table and the above explanations in the revision to ensure that readers can understand exactly how the AHP was conducted and how the priority rankings were obtained, thereby enhancing the transparency and reproducibility of our study’s prioritization process.

## Discussion

This mixed-methods, pre-implementation quality improvement (QI) study identified and prioritized key areas for enhancing type 2 diabetes mellitus (T2DM) care across 31 primary care facilities in Qatar. While not a comprehensive evaluation of all aspects of diabetes care, the study focused on a targeted set of 17 key performance indicators (KPIs) derived from national clinical practice guidelines adapted from the American Diabetes Association (ADA) and International Diabetes Federation (IDF) standards. These KPIs were prioritized using a Delphi technique, in which a panel of clinical experts rated their importance to guide subsequent audit and improvement planning.

The Delphi process in this study successfully achieved strong consensus among clinical experts regarding the importance of the 17 selected key performance indicators (KPIs) for type 2 diabetes care. Experts were asked to rate each KPI based on its perceived clinical importance in improving diabetes management within the primary care setting. Their ratings reflected their professional judgment, experience, and understanding of the local context, including the potential impact of each KPI on patient outcomes. However, it is important to clarify that the Delphi method, as applied here, was not designed to explicitly assess the feasibility of implementing each KPI in practice or to evaluate existing practice norms across centers. For example, while “lifestyle education” and “HbA1c testing” were rated as highly important, these scores do not necessarily indicate how easily these interventions can be operationalized within the current resource and workflow constraints of each health facility. Similarly, the observed variability in expert ratings for certain KPIs such as foot examinations or ASCVD risk assessments may reflect differences in clinical emphasis, perceived relevance, or contextual familiarity, rather than systematic differences in feasibility or routine practice. These interpretations remain exploratory, as feasibility and practice norms were not directly measured in this phase. Future implementation phases may benefit from incorporating structured assessments of feasibility such as stakeholder interviews, implementation readiness assessments, or feasibility scoring frameworks to complement expert consensus and guide prioritization more effectively.^12^

The clinical audit revealed substantial inter-center variability in adherence to several KPIs. While statistically significant differences were observed, these may reflect random variation rather than systematic inconsistencies in care delivery. Factors such as differences in staffing levels, documentation practices, electronic health record (EHR) usage, and local workflows may contribute to this variability.^13^ Understanding these contextual factors will be essential in tailoring future interventions to specific center needs. The findings reveal systemic gaps in preventive care and patient engagement, even though clear standards of care are outlined in clinical practice guidelines that align with international recommendations.

Our findings are consistent with broader patterns observed in other regional and international settings. In the United Arab Emirates, a clinical audit reported that more than half of patients had not received foot or eye examinations at baseline.^14^ Similarly, a United Nations Relief and Works Agency for Palestine Refugees in the Near East (UNRWA) audit across five Middle Eastern countries found that only 47.3% of patients had documented annual eye exams, with foot exams even less frequently recorded.^15^ A pooled analysis of nine national surveys in Latin America and the Caribbean revealed that fewer than 50% of people with diabetes received a foot exam in the past year, with some countries reporting rates as low as 12%.^16^ Even in high-income countries, gaps persist: a rural U.S. clinic reported a baseline foot exam rate of 43%, which improved to 74% following a nurse-led intervention.^17^ In the UK, the National Diabetes Audit reported foot risk surveillance coverage of 72.5% in 2019–20, indicating that even in well-resourced systems, a significant proportion of patients may not receive annual foot assessments.^18^

The audit also revealed significant inter-center variability, with some centers achieving near-perfect compliance and others performing poorly. For example, foot exam completion ranged from 0% to 95.6%, and ASCVD risk scoring from 0% to 100%. These variations in care delivery underscore the need for standardized protocols and cross-center learning. The discrepancies between technical tasks (e.g., laboratory ordering) and hands-on assessments (e.g., foot examinations) suggest that workflow design and role clarity may influence care delivery. Embedding clinical decision support tools in electronic medical records (EMRs), integrating preventive tasks into standard workflows, and leveraging team-based care (e.g., nurse-led foot checks) may help address these gaps. The low rates of ASCVD risk assessment, despite high lab completion, indicate missed opportunities to translate data into actionable risk stratification. Automated risk calculators and EMR prompts could support more consistent implementation.^19^

The low uptake of lifestyle education and individualized HbA1c goal setting in our audit mirrors findings from other studies. In the UAE, over 70% of patients had not received counseling on medication adherence or lifestyle modification.^14^ A 2019 audit in Qatar found that while 89.6% of patients received foot exams, only 39.9% were referred for dietary counseling.^20^ A multinational survey reported that 30% of patients were unaware of their HbA1c goals, and even among those who were aware, only 39.2% achieved them.^21^ These findings suggest that while clinical monitoring is often prioritized, patient engagement and shared decision-making remain underdeveloped in routine care.

Several statistical analyses were conducted to characterize the performance landscape and directly inform prioritization decisions in relation to the study objectives. Chi-square tests confirmed statistically significant inter-center variability across all 17 KPIs (p < 0.001), addressing the objective of identifying where and how widely care gaps occur. This variability implies that system-level interventions alone may be insufficient; center-specific strategies are likely needed to achieve uniform improvement. Paired t-tests quantified the magnitude of gaps between related indicators for example, foot exams lagged behind lab tests by a mean of 57.7 percentage points, and follow-up HbA1c testing was approximately 40 percentage points lower than baseline testing rates. These within-center differences help to distinguish structural deficiencies (where even centers with strong laboratory processes fail on hands-on assessments) from isolated documentation gaps, thereby guiding the selection of targeted intervention types. A moderate positive correlation between lifestyle education and dietitian referrals (r = 0.40, p = 0.024) suggests that patient-centered counseling may drive broader care coordination. This finding is clinically meaningful: interventions designed to strengthen lifestyle counseling may produce a beneficial cascade effect on referral pathways, yielding co-benefits beyond the primary target. Linear regression further identified lifestyle education as a significant predictor of dietitian referral rates (β = 0.524, 95% CI [0.07, 0.98], p = 0.025), whereas baseline lab completion was not (β = –0.163, p = 0.66). This differential predictive pattern suggests that patient-engagement processes rather than technical monitoring tasks are the primary drivers of integrated preventive care, reinforcing the high priority assigned to lifestyle education in the AHP analysis. However, the lack of meaningful correlation between most other indicator pairs implies that QI efforts must be multifaceted and tailored to specific domains, as high performance in one area does not guarantee excellence in others.

The Analytic Hierarchy Process (AHP) prioritized KPIs based on clinical impact, feasibility, and urgency. Indicators with high audit adherence, such as metformin initiation, received lower priority scores, likely due to their perceived lower urgency for improvement. This aligns with prioritization frameworks that emphasize targeting areas with the greatest potential for impact.^22^ The top-ranked indicators ASCVD risk assessment, foot examination, and lifestyle education reflect domains with both low adherence and high clinical relevance, making them suitable targets for initial intervention.

In summary, this pre-implementation QI study used a structured, mixed-methods approach to identify and prioritize key gaps in T2DM care across 31 primary care facilities in Qatar. Through the Delphi technique, practicing physicians rated the importance of 17 KPIs, fostering clinical ownership and alignment with frontline priorities. A clinical audit revealed wide variability in adherence, particularly in preventive care and patient engagement, reflecting random practice variation and contextual challenges. The AHP method further engaged clinicians in prioritizing interventions based on clinical impact, feasibility, and urgency. This participatory approach helped build a sense of responsibility and buy-in among clinical staff, which is essential for successful implementation and sustainability of future interventions.^23^

## Strengths

This study’s strengths include its targeted, system-wide scope and the integration of qualitative and quantitative methods. The Delphi process engaged a diverse panel of clinicians, ensuring contextual relevance and clinical validity of the selected KPIs. The baseline audit, involving 450 patients across all 31 PHCC centers, exceeded common audit sampling thresholds and provided a robust snapshot of care delivery. The use of inferential statistics added analytical depth, enabling identification of performance variability and actionable predictors. The AHP methodology introduced transparency and structure to the prioritization process, balancing clinical impact, feasibility, and urgency. Finally, the inclusion of a patient satisfaction survey, though modest in size, offered valuable insights into patient-reported experiences and highlighted communication gaps, particularly around goal setting.

## Limitations

Several limitations of this study should be acknowledged. First, it serves as a baseline assessment and does not measure the effect of interventions on clinical outcomes. Second, reliance solely on EMR documentation may lead to an underestimation of care provided if some actions went unrecorded. Third, the Delphi panel did not include patient representatives, which may have limited the breadth of perspectives. The patient survey was also limited in scope, with only 60 participants and simple yes/no responses, potentially missing the complexity of patient experiences. Although the AHP prioritization process was systematic, it relied on subjective judgments when weighting criteria and scoring feasibility and urgency. Nevertheless, the consistency between AHP outcomes, audit findings, and expert consensus suggests that the prioritization process was robust. While the study intentionally did not evaluate specific interventions, its primary aim was to create a reliable, consensus-based framework for prioritizing quality improvement actions. By emphasizing careful pre-implementation planning, the study seeks to ensure that future interventions are both evidence-based and suited to local conditions, thereby increasing their likelihood of successful implementation and real-world impact.

## Conclusions and Recommendations

This study identified critical deficits in the quality of primary diabetes care, particularly in cardiovascular risk management, foot disease prevention, and personalized glycemic goal setting, despite strong consensus on the importance of these interventions. The observed variability in care delivery across PHCC centers underscores the need for standardized protocols, targeted training, and system-level support.

Future research should evaluate the impact of these interventions on both process adherence and clinical outcomes and explore contextual barriers to implementation through qualitative inquiry. The Delphi–AHP framework may also be adapted for other chronic conditions or care domains, offering a scalable model for evidence-informed, data-driven quality improvement.

By focusing on the “vital few” gaps such as ASCVD risk assessment, foot exams, and patient education, primary care systems can make meaningful progress toward delivering more consistent, equitable, and guideline-concordant diabetes care. These efforts align with international calls for strengthening primary care in chronic disease management and contribute to the broader goal of improving population health outcomes.

To ensure accountability and measure the effectiveness of the implemented quality improvement plan, a structured re-evaluation will be conducted following its execution. This follow-up assessment will help determine the impact of the interventions and guide further refinements to sustain and scale improvements.

## Data Availability

All relevant data from this study will be made available upon study completion.*
